# Supplementary material for: Prostaglandin F2α regulates mitochondrial dynamics and mitophagy in the bovine corpus luteum
Source: Life Sci Alliance. 2023 May 15;6(7):e202301968. doi: 10.26508/lsa.202301968 (PMC10185813; doi:10.26508/lsa.202301968)

Figure 2: Source Data

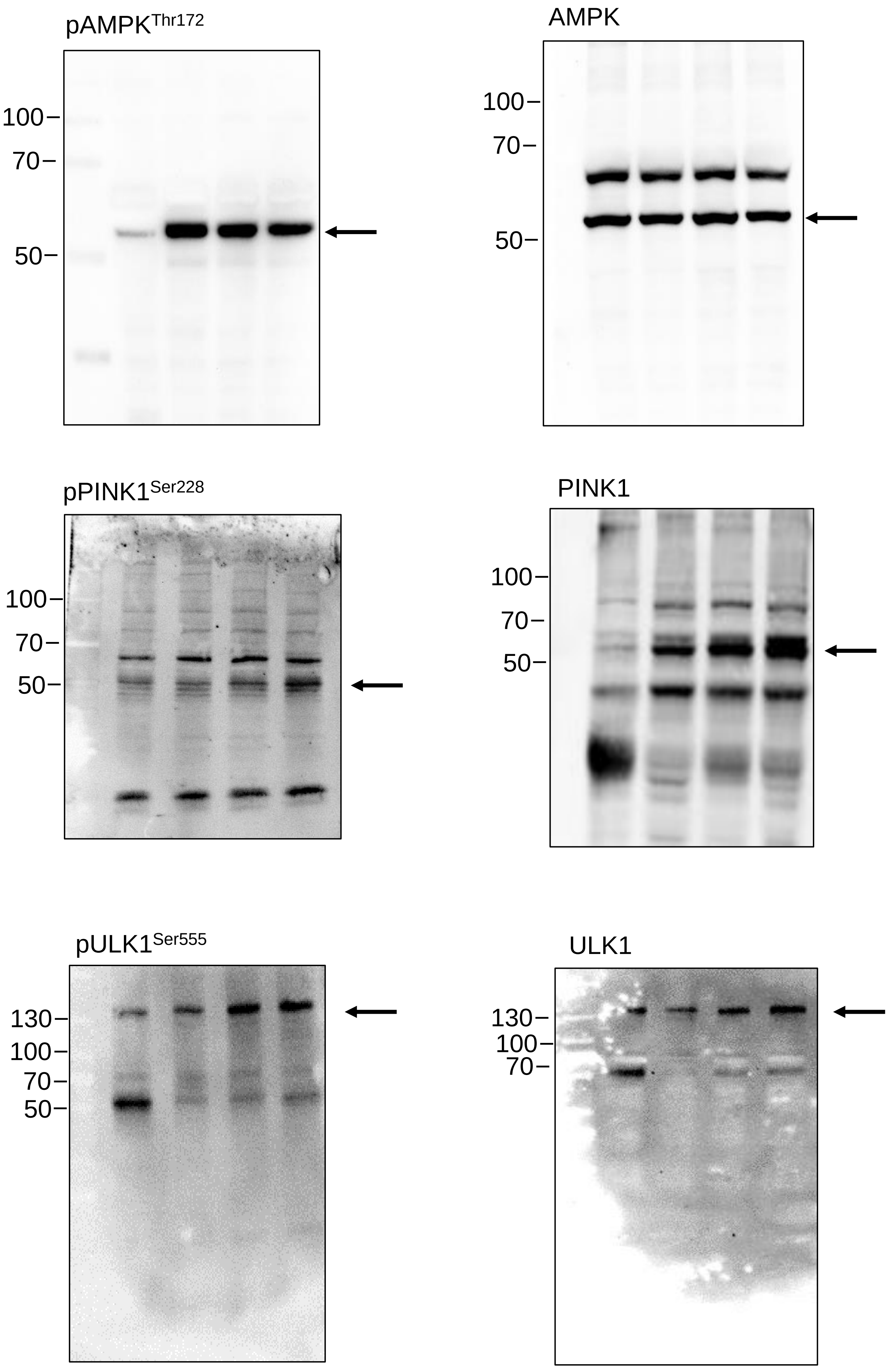

Figure 2 Cont.: Source Data

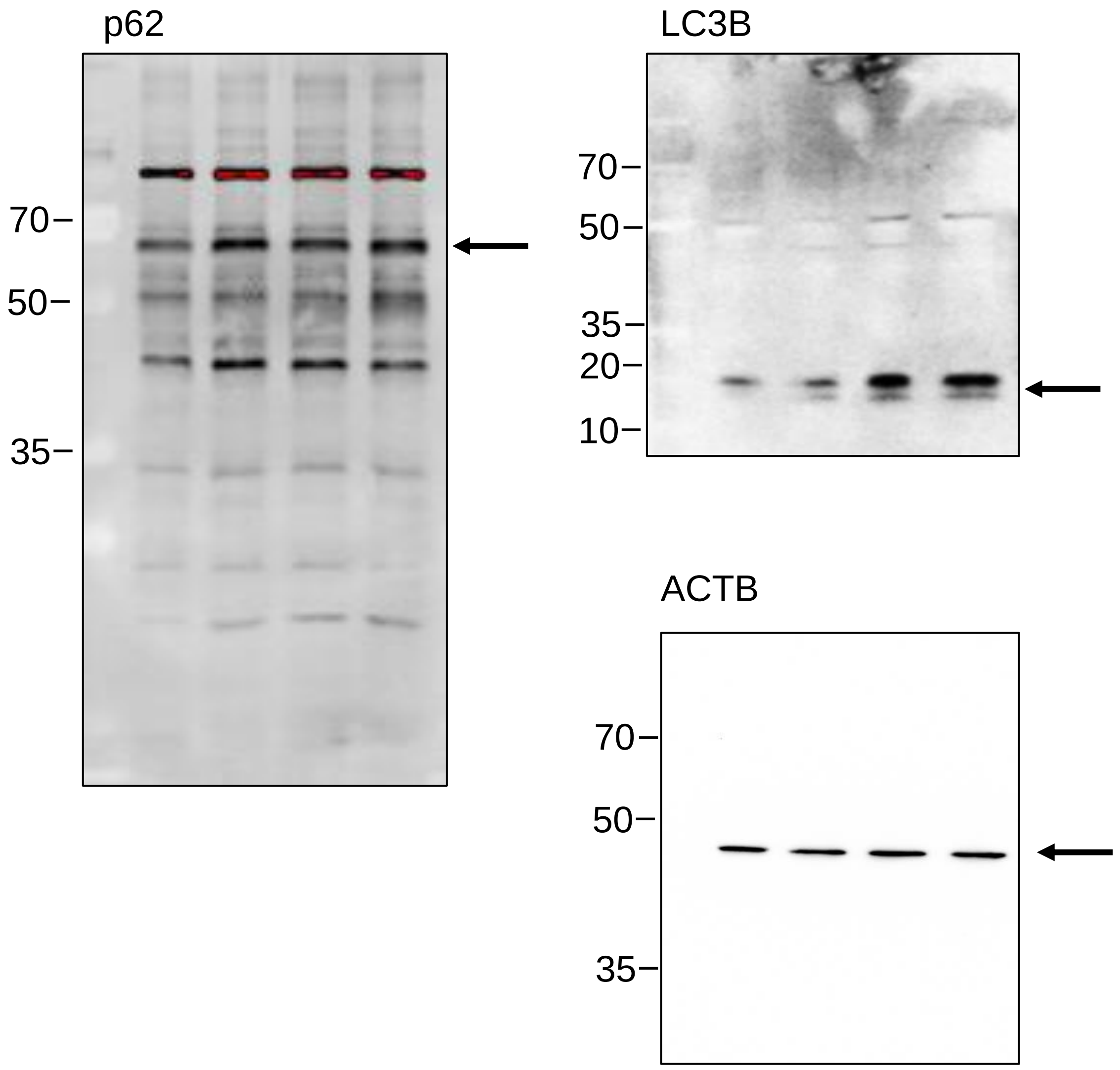

Figure 3A: Source Data

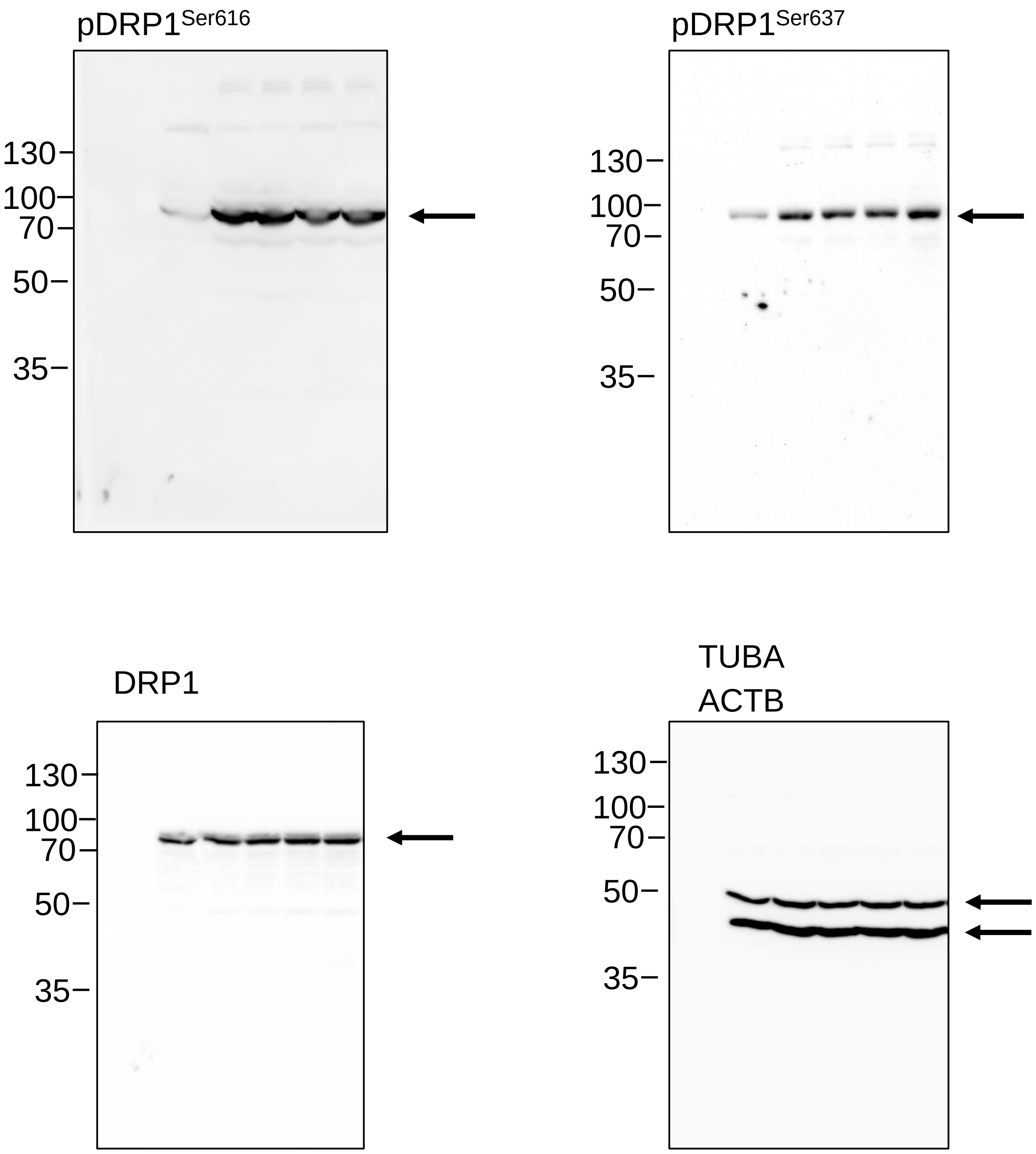

Figure 4E: Source Data

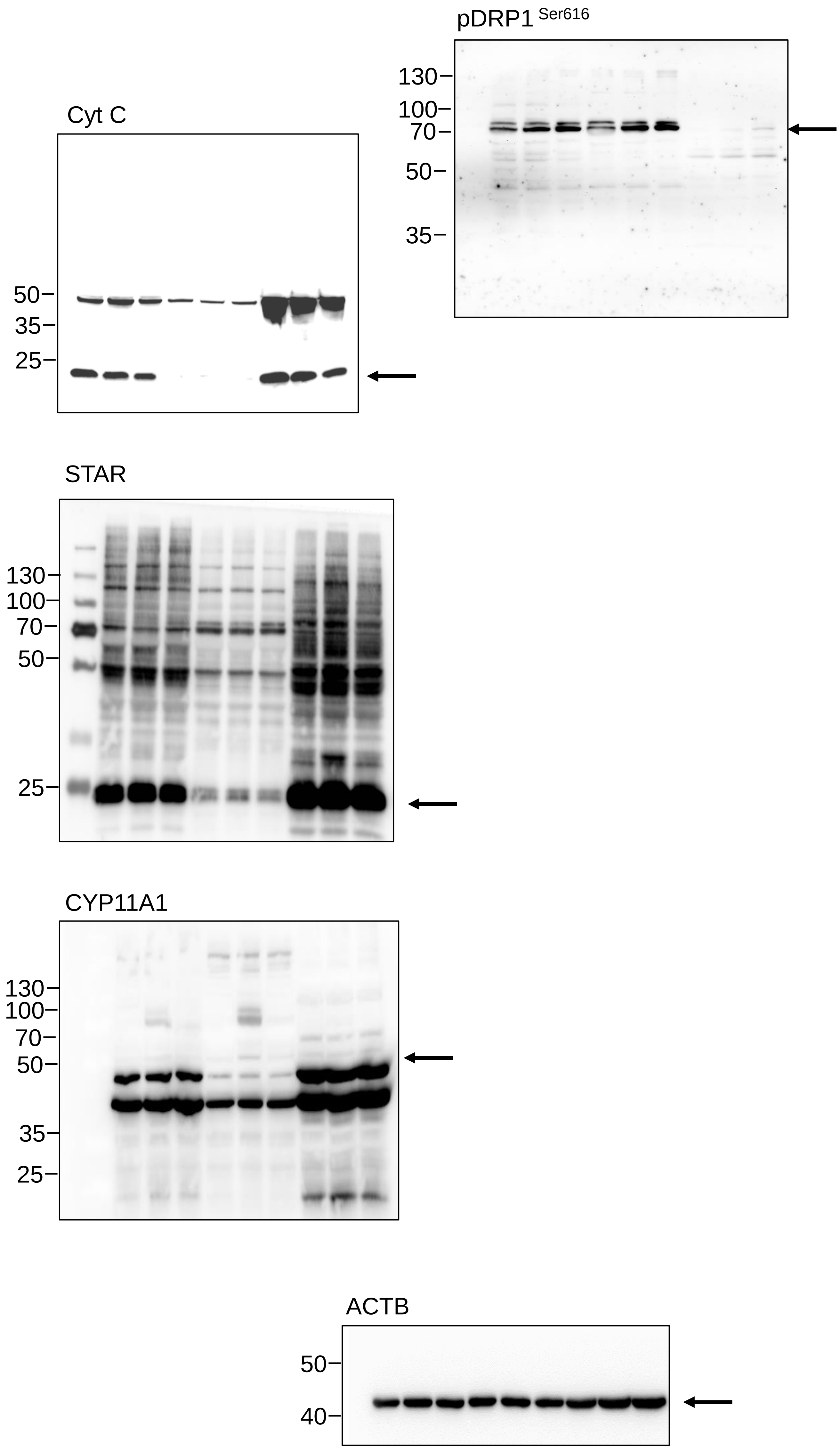

Figure 4E Cont: Source Data

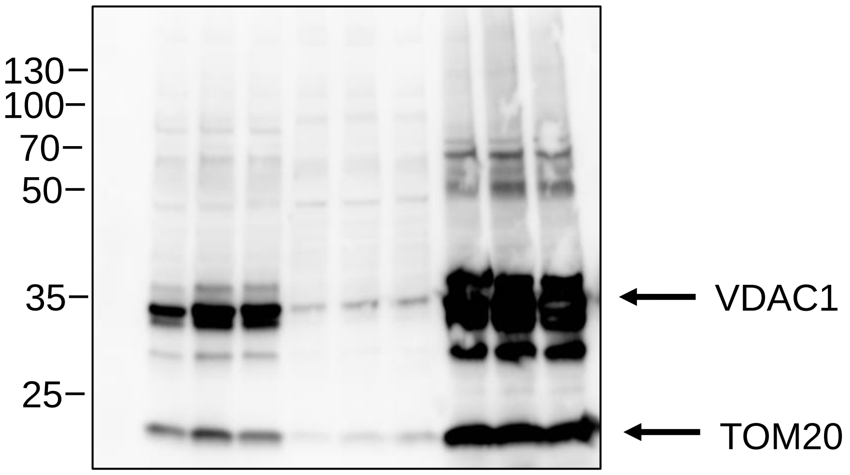

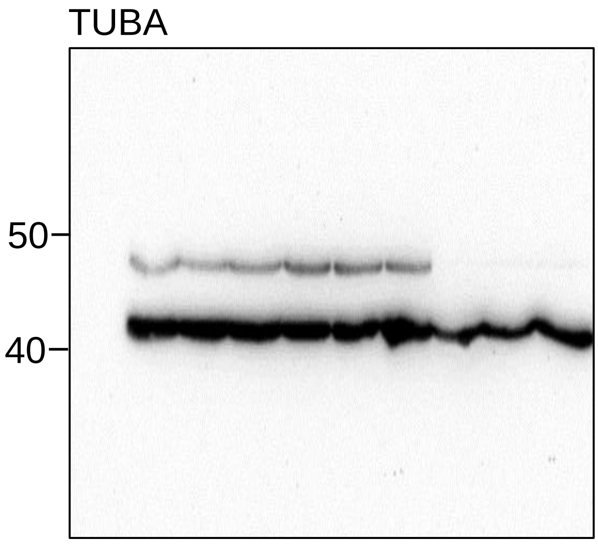

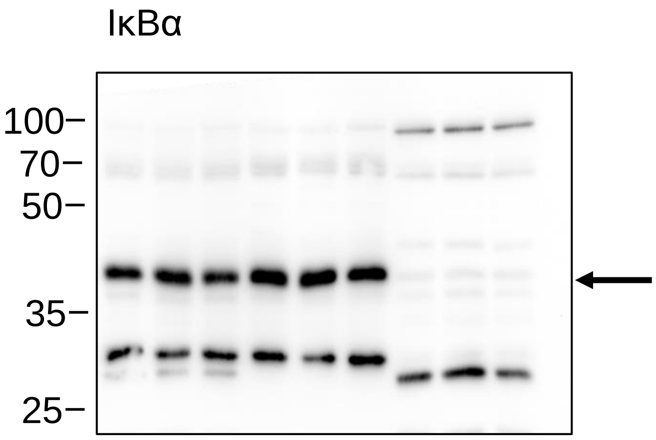

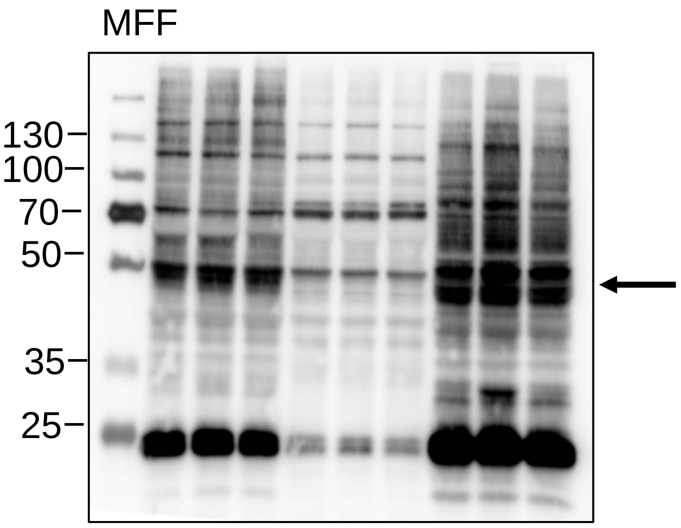

Supplement: Supplementary file 1 [file LSA-2023-01968_SdataF2_F3_F4.pdf]
